# Temporal patterns in acoustic presence and foraging activity of oceanic dolphins at seamounts in the Azores

**DOI:** 10.1038/s41598-020-60441-4

**Published:** 2020-02-27

**Authors:** Irma Cascão, Marc O. Lammers, Rui Prieto, Ricardo S. Santos, Mónica A. Silva

**Affiliations:** 1 Marine and Environmental Sciences Centre (MARE), Institute of Marine Research (IMAR) and Okeanos R&D Centre, University of the Azores, Rua Frederico Machado 4, 9901-862 Horta, Portugal; 2 Hawaiian Islands Humpback Whale National Marine Sanctuary, National Oceanic and Atmospheric Administration (NOAA), Kihei, HI 96753 USA; 3 Oceanwide Science Institute (OSI), PO Box 61692, Honolulu, HI 96744 USA; 4 Biology Department, Woods Hole Oceanographic Institution, Woods Hole, MA 02543 USA

**Keywords:** Ecology, Ocean sciences

## Abstract

Several seamounts have been identified as hotspots of marine life in the Azores, acting as feeding stations for top predators, including cetaceans. Passive acoustic monitoring is an efficient tool to study temporal variations in the occurrence and behaviour of vocalizing cetacean species. We deployed bottom-moored Ecological Acoustic Recorders (EARs) to investigate the temporal patterns in acoustic presence and foraging activity of oceanic dolphins at two seamounts (Condor and Gigante) in the Azores. Data were collected in March–May 2008 and April 2010–February 2011. Dolphins were present year round and nearly every day at both seamounts. Foraging signals (buzzes and bray calls) were recorded in >87% of the days dolphin were present. There was a strong diel pattern in dolphin acoustic occurrence and behaviour, with higher detections of foraging and echolocation vocalizations during the night and of social signals during daylight hours. Acoustic data demonstrate that small dolphins consistently use Condor and Gigante seamounts to forage at night. These results suggest that these seamounts likely are important feeding areas for dolphins. This study contributes to a better understanding of the feeding ecology of oceanic dolphins and provides new insights into the role of seamount habitats for top predators.

## Introduction

Animals should make optimal decisions about where and when to forage to maximize energy intake^1^ and it is expected that predators preferentially associate with areas where prey density is high^2^. Foraging activity of air-breathing diving predators like cetaceans is also severely constrained by the vertical distribution of their prey^3^. In the pelagic realm, where prey patchiness is usually high and topography deep, biophysical coupling at bathymetric and oceanographic features can aggregate prey at accessible diving depths. This “prey aggregator” effect has often been invoked to explain the association of cetaceans to static and dynamic features^4–6^.

Seamounts represent important discontinuity structures in the open ocean that may promote a range of physical processes that can serve to concentrate prey^7^. Upwelling in the vicinity of these structures can stimulate local productivity by bringing cool, nutrient-rich water into the photic zone. Enhanced water column mixing may also push weakly swimming zooplankton and larval/juvenile fish close to the surface. Seamounts may also be responsible for entrapping vertically migrating species within the depth range of predators^7–9^.

Acoustic monitoring at Cross seamount (350–450 m depth) near Hawaii, and at a seamount chain in the central equatorial Pacific (1300 m depth), detected echolocation signals from beaked whales on most recording days^10,11^. The authors hypothesized that seamounts increase foraging opportunities for beaked whales, by enhancing local prey concentration and facilitating prey capture^10^. In contrast, sperm whales (*Physeter macrocephalus*) were rarely detected at the equatorial Pacific seamount chain^11^ and their seasonal presence at Kelvin seamount (~1600 m) appears to be related to regional variations in primary productivity^12^. Satellite telemetry studies showed that humpback whales (*Megaptera novaeangliae*) spent several days around the Antigonia seamount (60 m) and Torche Bank (30 m), off New Caledonia^13^, while North Atlantic blue whales (*Balaenoptera musculus*) occasionally engaged in area-restricted search (ARS) behaviours in the deep waters (>5000 m) around New England Seamounts^14^. In a similar study also based on satellite telemetry data, blue and fin (*B. physalus*) whales also engaged in ARS behaviour along a chain of shallow seamounts off the Azores^15^.

One of the first studies to specifically investigate the effect of seamounts on the distribution of cetaceans was conducted in the Azores. This work demonstrated that common dolphins (*Delphinus delphis*) were significantly more abundant in the vicinity of some shallow water seamounts, while the relative abundance of spotted dolphins (*Stenella frontalis*), bottlenose dolphins (*Tursiops truncatus*) and sperm whales was not associated with the presence of seamounts^8^. More recently, Tobeña *et al*.^16^ developed habitat suitability models for 16 cetacean species in the same area. While presence and depth of seamounts had no effect on the distribution of any cetacean species, the density of seamounts (number of seamounts/km^2^) was a significant predictor of the distribution of sperm whales, killer whales (*Orcinus orca*), common and spotted dolphins.

Hence, the relationship between cetaceans and seamount habitats remains elusive. This is not surprising given that most research on cetacean usage of seamount habitats has relied on short-term observations and has often focused on seamounts for which there is little knowledge about the distribution and abundance of pelagic communities.

**Figure 1:**
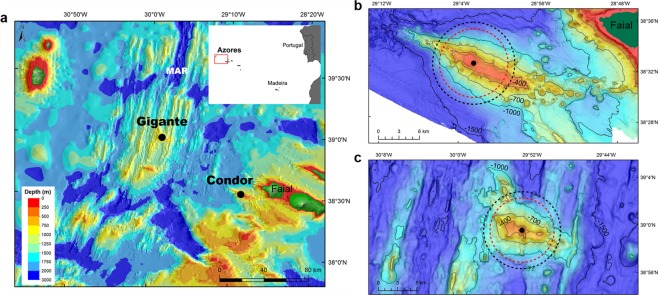
Study area. Location of the Ecological Acoustic Recorders (EARs) (black dots) at Condor and Gigante seamounts (**a**), in relation to the islands of the Azores and the Mid-Atlantic Ridge (MAR). Detailed map of Condor (**b**) and Gigante (**c**) seamounts. Maximum detection range of dolphin whistles (6 km; dashed black circle) and of dolphin clicks (5 km; dashed red circle) are shown. Warmer colours indicate shallower depths. Figure produced with ArcGIS 10.1 (http://www.esri.com). Bathymetry data credits: Azores^70^; Condor - EMEPC, DOP/UAz, Project STRIPAREA/J.Luís/UAlg-CIMA; Gigante - EMEPC, IMAR-DOP/UAz.

Here we use a long-term dataset from passive acoustic recorders deployed at two seamounts in the Azores, Condor and Gigante (Fig. 1), to investigate the relationship of small delphinids to these habitats. Condor and Gigante are two relatively small, shallow-intermediate seamounts (summits at ~190 m depth) that rise more than 1000 m from the surrounding seafloor. Active acoustic surveys conducted at these seamounts found very high densities of micronekton (i.e., assemblages of small (<20 cm) pelagic fish, cephalopods and crustaceans^17^) over the plateau of both seamounts, throughout the day, seasons and years^18^. Such high densities are believed to be caused by the presence of a seamount-associated micronekton community and by the retention of vertically migrating micronekton at the summits^19^.

Five species of small dolphins occur in the Azores. Common and spotted dolphins are by far the most frequent and abundant, comprising over 45% of all cetacean sightings, followed by bottlenose and Risso’s dolphins (*Grampus griseus*); striped dolphins (*S. coeruleoalba*) are sighted only sporadically^20^. These dolphin species forage opportunistically on a variety of epipelagic and mesopelagic schooling fishes and cephalopods, key constituents of the micronekton^21–24^. They produce a variety of high-intensity acoustic signals making them ideal for monitoring with passive acoustics^25,26^. Detection of acoustic signals mainly used by dolphins when foraging (such as buzzes and bray calls) can offer unique insights into the foraging activity of dolphins^27–30^ at seamount habitats.

This study investigates the temporal dynamics of dolphin acoustic detections and behaviour in Condor and Gigante seamounts to understand (i) how dolphins use seamount habitats, and (ii) if and how seamounts affect dolphin foraging behaviour. Our results indicate that dolphins use these seamounts intensively during the whole year to forage at night, possibly to take advantage of increased densities of micronekton prey within their foraging depths.

## Results

The Ecological Acoustic Recorders (EARs) on Condor and Gigante seamounts produced 72,660 data files, corresponding to 689 unique days and 1,467 total hours of recordings (Table 1). Dolphins were detected 16,945 times, which is equivalent to 11.5 detections/hour. Overall, 35% of recordings contained foraging signals (buzzes and/or bray calls), 36% echolocation clicks with social signals (whistles and/or burst-pulsed sounds), 21% only social signals and 8% only echolocation clicks.

**Table 1:** Summary of acoustic recordings on Condor and Gigante seamounts.

Location	Period	Duty cycle	Sensitivity	N° days	N° hours
Condor	Mar–May 2008	30 s/10 min	−193.14	75	88.6
	Apr–Sep 2010	90 s/15 min	−194.17	159	379.8
	Sep 2010–Feb 2011	90 s/15 min	−194.17	153	366.1
**Total**				**387**	**834.5**
Gigante	Mar–May 2008	30 s/10 min	−193.64	72	85.7
	Jul–Sep 2010	90 s/15 min	−193.14	71	167.6
	Sep 2010–Feb 2011	90 s/15 min	−193.14	159	379.8
**Total**				**302**	**633.1**

The table indicates the time period, duty cycle (sec on/min off), hydrophone sensitivity (dB re 1 V/μPa), number of days and hours of recordings of each deployment.

### Dolphin acoustic presence

**Figure 2:**
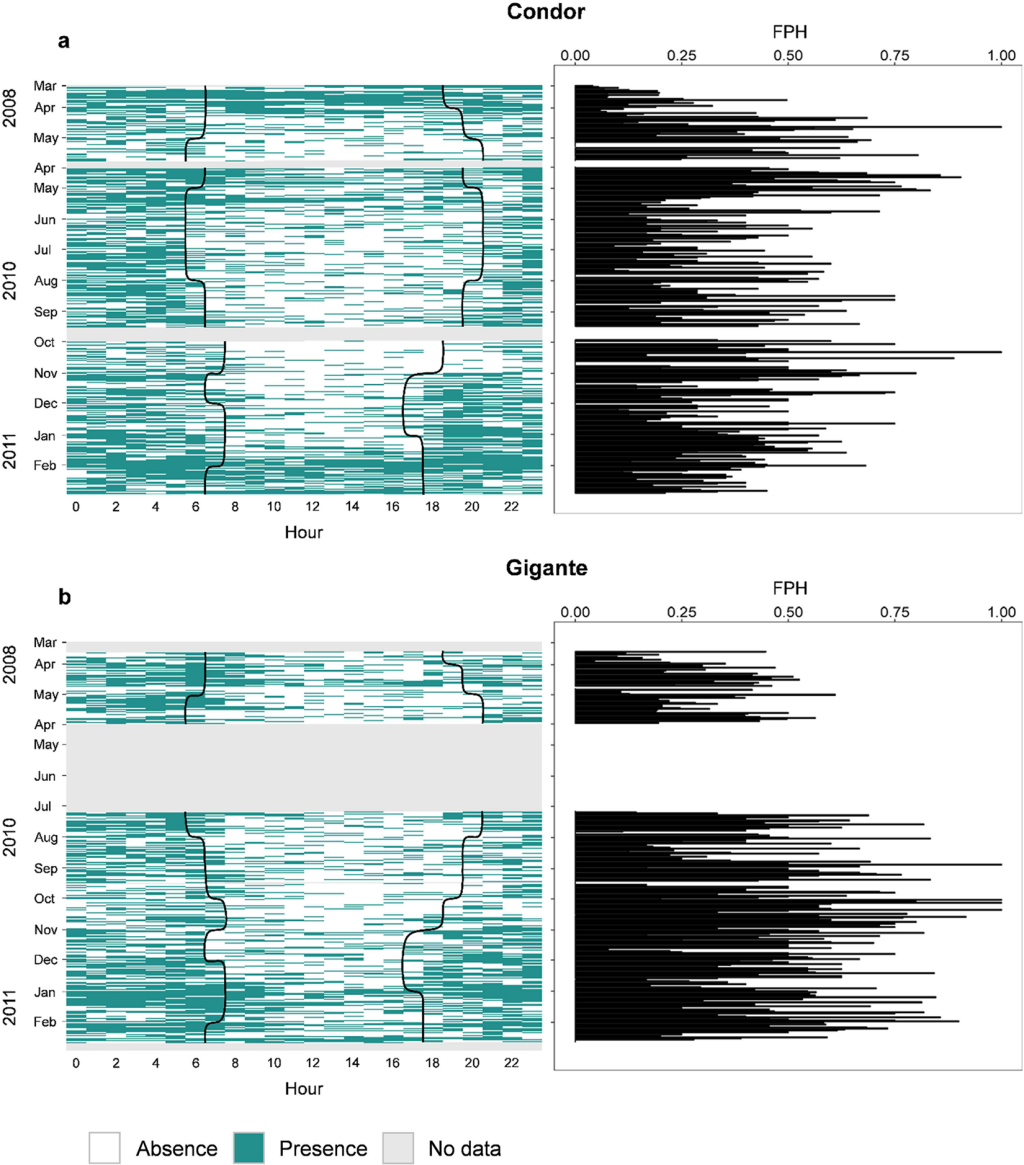
Daily dolphin detections per hour of day (left plots) and daily proportion of dolphin foraging positive hours (FPH) (right plots) in Condor (**a**) and Gigante (**b**) seamounts. Each rectangular cell in the left plots represents acoustic presence (green) or absence (white) for each hour and day of the study period. Periods of missing data are shown in grey. Vertical black lines indicate sunrise and sunset times.

**Figure 3:**
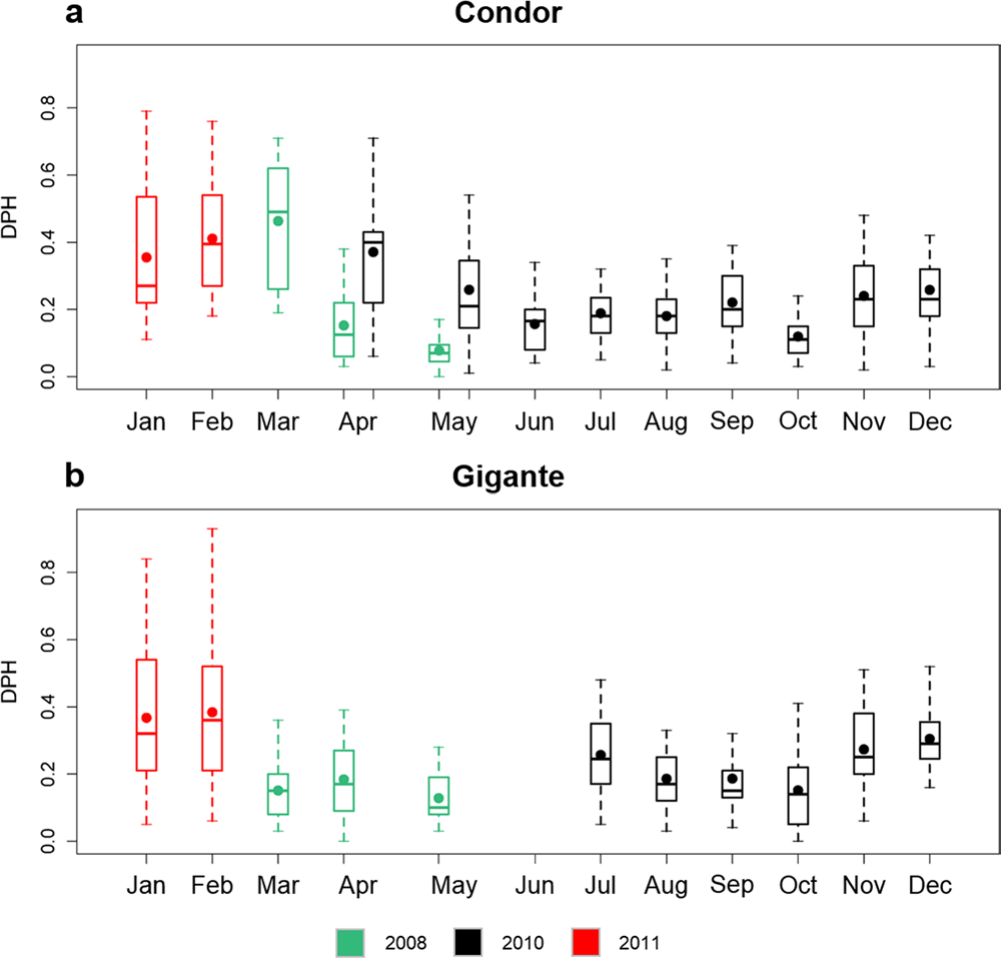
Daily proportion of dolphin positive hours (DPH) per month and year of sampling in Condor (**a**) and Gigante (**b**) seamounts. Box colour indicate the years of sampling (green: 2008, black: 2010, red: 2011). Box plots show the mean (dots), median (bars), quartiles (box), and 1.5 times interquartile range or extreme values (whiskers).

Dolphin vocalizations were detected in all years, months and nearly every day at both seamounts. Detections occurred in 99.5% and 98.7% of the recording days at Condor and Gigante, respectively (Fig. 2). The proportion of dolphin positive hours per day (DPH) did not differ between the two seamounts but varied significantly across sampling periods (Supplementary Table S1). When comparing the sampling periods with recordings available for both sites, temporal variability in daily presence was similar in Condor and Gigante: mean DPH was highest in January and February 2011, and in March 2008 (only in Condor), intermediate in November and December 2010, and lowest in July–October 2010 and in April–May 2008, although differences in Gigante were not always significant (Fig. 3, Supplementary Table S3). In Condor, daily presence was higher in April and in May 2010 than in April and May 2008 (Fig. 3, Supplementary Table S2).

**Figure 4:**
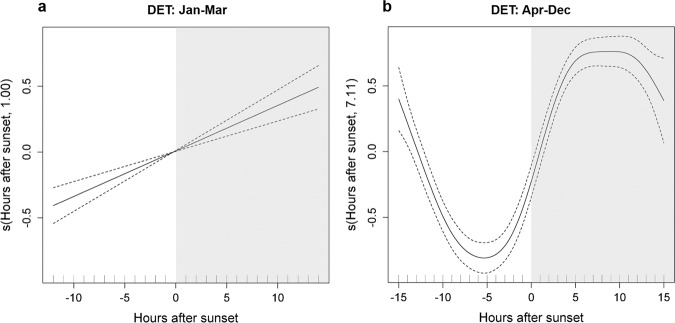
Diel patterns in dolphin detections at Condor and Gigante seamounts. Response curves of the GAMM model for presence/absence of dolphin detections relative to hours after sunset in two periods: January–February 2011 and March 2008 (**a**) and April–May 2008 and April–December 2010 (**b**). The solid line represents the smoother estimated by the GAMM and dashed lines denote the approximate 95% confidence intervals. Estimated degrees of freedom (edf) of the smooth is displayed on the y-axes. Tick marks on the x-axis show sample values. Night periods (shaded area) correspond to positive hours after sunset and day periods (white area) to negative hours.

Dolphins were detected both day and night at Condor and Gigante seamounts, but the majority of detections occurred during the night (Fig. 2). Day-night differences in dolphin detections were especially pronounced in June-December 2010, a pattern observed at both seamounts, despite data gaps Gigante. In January-February 2011 and in March 2008, there were more detections during the day, and diel differences were not as evident. These results are supported by the Generalized Additive Mixed Model (GAMM), which revealed that dolphin detections were significantly related with hours after sunset, but the relationship differed between the two periods analysed (Fig. 4, Supplementary Table S4). In January-March, dolphin detections increased linearly with increasing hours after sunset (Fig. 4a). In the remaining sampling period detections exhibited a clear diel rhythm: they were lowest 7–4 h before sunset, increased sharply 2 h prior to sunset peaking at 5 h after sunset, and decreased again 10–11 h after sunset (Fig. 4b).

### Dolphin foraging activity

Foraging activity was analysed using only the days and hours during which dolphins were detected. Foraging signals were recorded in 87% and 91% of the days that dolphins were detected at Condor and Gigante seamounts, respectively (Fig. 2). The Generalized Linear Model (GLM) model showed that the proportion of foraging positive hours per day (FPH) did not vary significantly between seamounts nor among sampling periods (Supplementary Table S1). On average, dolphins foraged 3.9 ± 2.7 (s.d.) hours per day at Condor and 4.8 ± 3.2 hours per day at Gigante, with a maximum of 19 and 16 hours at Condor and Gigante, respectively.

**Figure 5:**
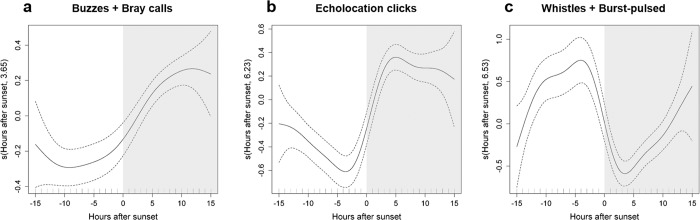
Diel patterns in dolphin acoustic signals at Condor and Gigante seamounts. Response curves of the GAMM models for presence/absence of (**a**) buzzes and/or bray calls (foraging signals), (**b**) echolocation clicks and (**c**) whistles and/or burst-pulsed signals (social signals) relative to hours after sunset. The solid line represents the smoother estimated by the GAMM and dashed lines denote the approximate 95% confidence intervals. Estimated degrees of freedom (edf) of the smooth is displayed on the y-axes. Tick marks on the x-axis show sample values. Night periods (shaded area) correspond to positive hours after sunset and day periods (white area) to negative hours.

To investigate if time of year also influenced diel patterns in dolphin foraging activity and acoustic behaviour, we included in the GAMM models a different smoother for hours after sunset for each period (January-March and April-December) (not shown). Because the shape of the smoothers for different periods was remarkably similar, we chose to fit the models again using a single smoother for all data pooled. All classes of acoustic signals showed a very pronounced diel pattern (Fig. 5, Supplementary Table S4). Foraging signals and echolocation clicks were recorded predominantly at night (Fig. 5a,b). Foraging activity was low throughout the day, increased gradually during the afternoon and night until reaching highest values ~9 h after sunset (Fig. 5a). Echolocation activity was lowest 3 h prior to sunset, increased rapidly towards sunset, peaked 4 h after sunset and decreased again 11 h after sunset (Fig. 5b). Social signals were recorded more often during daylight hours than at night, with a clear peak in the afternoon (~4 h prior sunset) and subsequently decreasing (Fig. 5c).

## Discussion

This study shows that small dolphins consistently use Condor and Gigante seamounts to forage at night. Dolphins were acoustically detected in all years, months and nearly every day for which data were available, and engaged in foraging activity in the great majority of those days (Condor: 87%, Gigante: 91%).

Because we lacked data from all the months in any given recording year, monthly patterns and inter-annual changes in acoustic presence of dolphins at these seamounts could not be conclusively resolved. Nevertheless, our results show that the daily proportion of dolphin positive hours varied between months within years (in Condor and Gigante), as well as between the same months in different years (in Condor). Some of this intra- and inter-annual variability was related to the diel pattern in dolphin detections, which varied through the study period. In fact, DPH peaked in January–February 2011 and March 2008 (in Condor), when daytime detections were higher.

The variation in daily detection rates within and between years likely reflects changes in the number of dolphins and/or time spent at these seamounts. Changes in seamount use by dolphins could be driven by local shifts in prey availability and foraging conditions or reflect changes in the distribution of dolphins at broader spatial scales. A better understanding of the temporal habitat use patterns could provide further insights into the driving factors. Dolphin detections may also have been influenced by varying sound propagation conditions and ambient noise levels^31^.

The diel pattern in dolphin acoustic presence was evident at both seamounts, with higher rates of detections during nighttime periods. Although we cannot completely rule out the existence of periodic daily movements outside the EARs detection range, analysis of the acoustic behaviour of dolphins indicates a diel cycle in the use of distinct vocalizations, which may explain observed day-night differences in dolphin detections.

All categories of dolphin signals were detected both during the day and night at both seamounts but some signals were more frequently recorded during the day and others at night. Foraging signals (buzzes and/or bray calls) and echolocation clicks were lowest during daylight hours and increased around sunset, remaining high until much later in the night. In contrast, social signals (whistles and/or burst-pulsed) were mainly recorded during daylight hours, with detections decreasing sharply after sunset and increasing gradually throughout the night.

Similar diel cycles in vocal behaviour have been reported in several dolphin species. In general, whistling used in social interactions is higher during daylight hours or remain constant throughout the day, whereas echolocation clicks used primarily for foraging and navigating occur mainly at night^32–35^. In addition, concurrent observations of behaviour and acoustic recordings indicate that dolphins echolocate at high rates while foraging, exhibit low vocal activity while resting, and moderate vocal activity while socializing and travelling^32,36,37^. Increased vocal activity during nighttime foraging and lower vocal activity during socializing could explain the diel pattern in dolphin detections at seamounts.

High rates of nighttime foraging and echolocation in dolphins living in pelagic waters have been associated with feeding on vertical migrating micronekton organisms at night^32,34,35,38^. Most micronektonic taxa undergo diel vertical migrations, residing in deeper waters during the day, swimming towards the surface around sunset to feed during night, and returning to deeper waters at sunrise^39^. The depth distribution and density of prey, in addition to overall abundance, are critical to the foraging success of air-breathing diving predators because they determine whether prey items are accessible, how many prey can be encountered during a foraging dive and at what cost^40^. In order to minimize oxygen use during diving and maximize feeding rates, dolphins foraging over deep waters may benefit from concentrating their foraging activity at night, when suitable concentrations of vertically migrating prey are available within their diving range. Hawaiian spinner dolphins (*Stenella longirostris*) have been shown to track the diel vertical migration of the deep scattering layer (DSL), and to forage mainly at night, when the layer is shallower^41,42^. Melon-headed whales (*Peponocephala electra*) rest and socialize over shallower waters during the day, and move towards deep water to feed at night on mesopelagic prey^43^, with a concurrent increase in echolocation click rates and less whistling^34^.

Dolphins foraging at Condor and Gigante seamounts could benefit from increased foraging opportunities during the day, compared to dolphins foraging over deep waters. Daytime micronekton densities over the plateaus of Condor and Gigante are significantly higher than densities measured in open-waters, at any time of the day and across all seasons^18^. Micronekton layers are also strongly structured during the day, forming small patches less than 16 m thick, nested into layers extending 111 m vertically. These layers tend to remain close to the seafloor throughout the day (mean population centred at ~190 m)^19^. Although the exact composition of these micronekton layers is unknown, they presumably include small pelagic and benthopelagic fishes (*Anthias anthias*, *Callanthias ruber*, *Trachurus picturatus*, *Macrorhamphosus scolopax*, *Capros aper*), as well as mesopelagic organisms from the DSL that became entrapped over the seamount plateaus^19^.

Small dolphins are capable of diving to 200–300 m depth^44^ so the daytime depth of the micronekton layer over seamount plateaus is within the diving range of these dolphins. Therefore, we expected dolphins would take advantage of these dense prey patches close to the seamount plateau to forage throughout the day. If this were true, we would also expect the diel cycle in their foraging activity to have been less pronounced. Instead, our results revealed a strong diel pattern in foraging activity, with increasing levels just before darkness, high levels through the night and the lowest levels during the day, similarly to what has been reported in other habitats. This pattern coincided with an increase in acoustic density of prey in surface layers over Condor and Gigante seamount summits and slopes, resulting from the vertical migration of part of the DSL^19^. Dolphins’ foraging activity increased when prey from the DSL begins moving towards the surface and reaches the peak when these prey disperse in the upper water column.

It is possible that the benefits of foraging during the day on high-density prey aggregations near the seafloor may be offset by the costs of diving deeper, so dolphins choose to wait for the prey to be available at shallower depths. Even if the daytime depth of the micronekton layer is accessible to these dolphins, the longer and deeper dive may imply greater energy expenditure^45^ and more time to recover oxygen stores at the surface^46^, which translates into lower feeding rates. It comes to a point when the energetic gains are offset by the costs associated with acquiring energy, and foraging is no longer an optimal strategy^47^. This explanation is consistent with a recent study on humpback whales that showed that whales increased their foraging effort at night when prey was shallowest but less densely distributed, presumably to minimize diving and searching costs^48^. Prey depth is also known to be a strong predictor of the habitat use of seabirds and seals^3^.

Alternatively, the diel cycle in detection of foraging signals could reflect a switch from acoustic to visual predation. Given the daytime depth of micronekton (~190 m) is deeper than the lower limit of the photic zone (~150 m)^49^, it seems unlikely that dolphins would rely primarily on vision for detecting prey.

## Conclusion

Passive acoustic monitoring at two seamounts in the Azores shows that oceanic dolphins frequently foraged in these seamount habitats during the night. Dolphins were detected nearly every day and foraging activity occurred on most recording days. Dolphin foraging activity at Condor and Gigante seamounts was significantly higher at night than during the day, similarly to what was reported for dolphins foraging in coastal areas and in pelagic deep waters. This is somewhat surprising and suggests that dolphins did not exploit the high densities of micronekton prey that are continuously available to them near the plateau (~190 m depth) of these seamounts. Instead, foraging activity increased shortly after sunset, coinciding with the upward migration of organisms from the DSL. The benefits of exploiting higher prey densities may be offset by the costs of foraging at higher depths.

The depth rating of our acoustic system did not enable monitoring open-ocean areas to compare dolphin detections with seamount habitats. This would help understanding the ecological relevance of the high dolphin detection rates reported for Condor and Gigante seamounts, and clarifying whether oceanic dolphins specifically target these seamounts to forage. Studies using other acoustic systems may provide these answers in the future, contributing to a better understanding of the role of seamount ecosystems for these predators.

Finally, this work emphasizes the bottom-up perspective but it will be equally important to understand the top-down effect of dolphins on the structure and functioning of seamount systems. Daily presence and regular foraging of dolphins at seamounts suggests these animals may play an important role in driving local dynamics of micronekton prey, regulating food web structure and composition, and in nutrient cycling^50,51^.

## Methods

### Data collection

Passive acoustic monitoring data were collected at two shallow-intermediate seamounts, Condor and Gigante, in the Azores (Fig. 1). Condor has a relatively flat summit with two major peaks at 182 m and 214 m depth and a total surface area of 11.6 km^2^. It is an elongated feature approximately 26 km long and 7.4 km wide at the 1000-m depth contour (Fig. 1b). Gigante reaches 161 m depth, and has a small plateau of 0.7 km^2^. It is about 16 km long and 6–13 km wide at the 1000-m depth contour (Fig. 1c)^18^.

At each seamount, an Ecological Acoustic Recorder (EAR) was moored ~10 m above the seafloor, at approximately 190 m depth. The EAR is a microprocessor-based autonomous recorder produced by Oceanwide Science Institute (Honolulu, HI)^52^ with a Sensor Technology SQ26-01 hydrophone that has a flat frequency response (±1.5 dB) from 18 Hz to 28 kHz with a sensitivity between −193 and −194 dB re 1 V/μPa (Table 1).

Acoustic recordings were collected in March-May 2008 and April 2010-February 2011, but there were periods with no data available due to gaps in successive deployments and occasional instrument failure (Table 1). Additionally, recorders had to be duty cycled. In the first deployment, the EARs were programmed to record 30 s every 10 min at a sampling rate of 50 kHz, providing an effective recording bandwidth of 25 kHz at a 5% duty cycle (Table 1). To increase the likelihood of recording dolphin vocalizations, the duty cycle was subsequently changed to record 90 s every 15 min (10% duty cycle). With this duty cycle, the system was capable of storing data for periods of 4 to 6 months. After this period, the EARs were recovered to download the data.

## Data analysis

### Data processing

**Figure 6:**
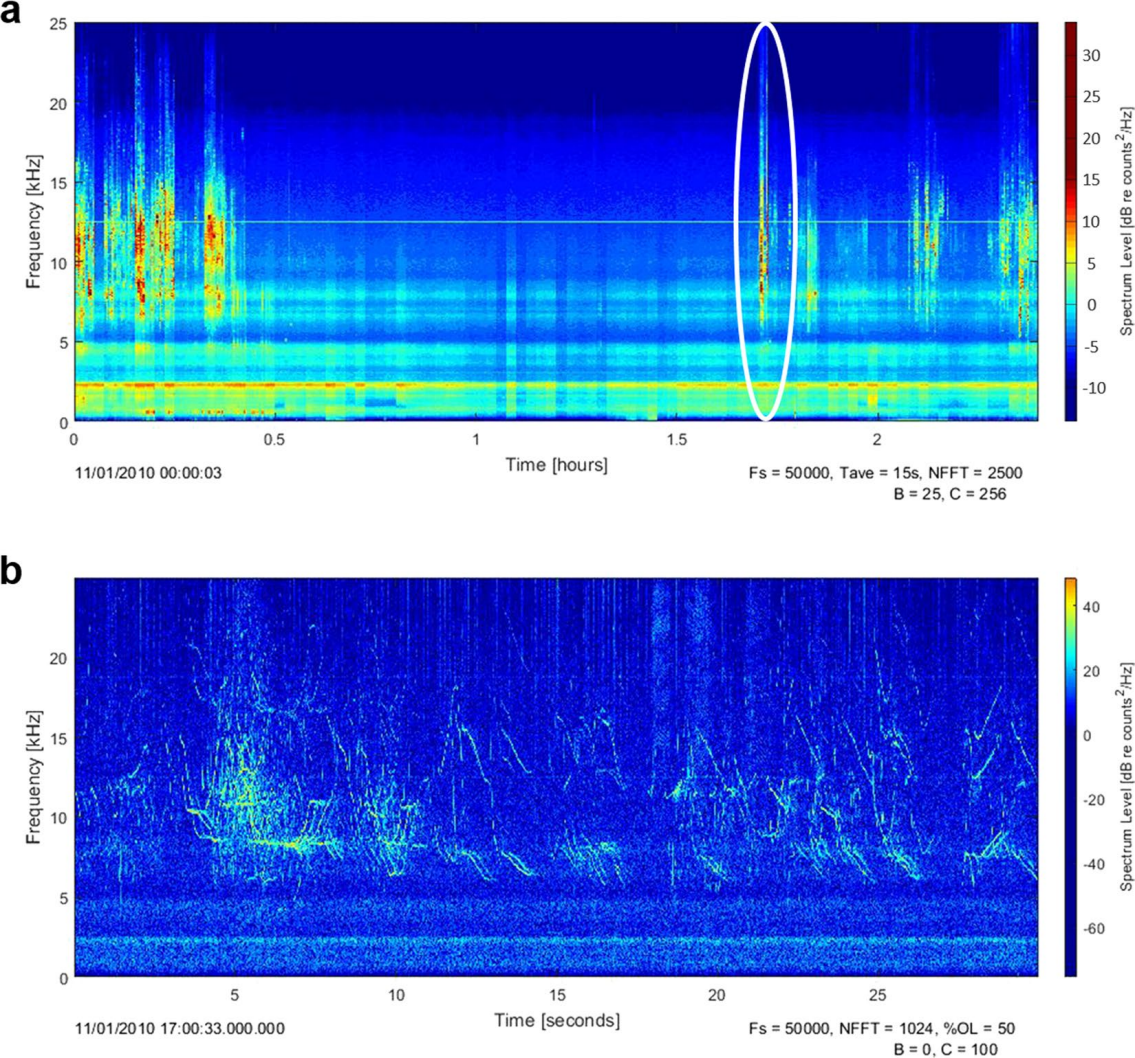
Long-term spectral averages (LTSA) and spectrogram. Plot a is an example of a 2.4-hour LTSA, corresponding to a day of recordings on Gigante seamount. Plot b is a spectrogram showing 30 s of data where you can observe dolphin whistles, clicks, and burst-pulsed signals. Warmer colours indicate the stronger intensity of sound (dB re 1 μPa^2^/Hz).

Acoustic data were processed using Triton, a custom Matlab script^53^. Triton was used to create long-term spectral averages (LTSA) of the acoustic recordings, which provide a means of quickly evaluating long-term data sets for acoustic events. Instead of inspecting short duration spectrograms for individual calls, successive spectra are calculated and averaged together. These averaged-spectra are arranged sequentially to provide a time series of the spectra^54^. Using LTSAs, delphinid whistling and echolocation clicking bouts can easily be distinguished from background noise and other biotic or abiotic sound sources (Fig. 6).

LTSAs were calculated with 20 Hz frequency and 15 s time resolution. These LTSAs were manually scrutinized to assign ones or zeros, representing presence or absence of dolphin signals, for every 1 h long LTSA segment. When dolphin signals were detected within a 1 h LTSA segment, 30% of the files with the strongest signals (indicated by dB intensity) were selected for visual inspection of the spectrograms to classify dolphin vocalizations and detect potential foraging activity.

Delphinid echolocation clicks are short, broadband pulses with peak frequencies that vary from tens of kilohertz to well over 100 kHz. These clicks generally occur in trains containing few to hundreds of clicks and are used for navigation and target detection and discrimination^55^. During the final stages of prey capture, these echolocation click trains become more rapid with shorter inter-click intervals forming buzzes^28,30^. Burst-pulsed signals are short, discrete bursts of broadband sound pulses believed to function mainly in social interactions^56^, although some signals may also play a role in foraging events, such as bray calls^27,29^. Bray calls consist of a single- or multi-unit sequences of sounds with burst-pulsed signals alternating with short tonal sweeps, only recorded for bottlenose dolphins^57–59^. Whistles are continuous, narrow band, frequency modulated signals that range in duration from several tenths of a second to several seconds. The fundamental frequency of most whistles ranges from 2 to 30 kHz. Whistles are believed to function in social contexts^60^. Based on the above, detection of buzzes or bray calls was used as an indicator of dolphin foraging activity^27–30^, and detection of any signal attributed to dolphins was used to indicate dolphin presence.

Delphinid species identification from acoustic data is challenging, due to the strong similarity of most signals, especially echolocation clicks. Because the sampling rate used in this study (50 kHz) did not allow us to record the full spectrum of echolocation clicks, we did not attempt to separate signals into probable species and pooled all acoustic data under a single delphinid group. Nevertheless, of the five dolphin species that occur in our study area^20^, Risso’s dolphin clicks have peaks frequencies slightly higher than frequencies recorded by our system^61^. Moreover, in the Azores, Risso’s dolphins show a preference for habitats close to the islands^16^ and striped dolphins are only occasional visitors^20^. Hence, we assume the large majority of acoustic detections to be of common, spotted and bottlenose dolphins.

We were unable to determine the detection range of the EAR, which may vary considerably, depending on location of the recorders, environmental conditions, as well as vocalization type and behavioural context^31,62,63^. However, given that the maximum expected detection range of dolphin clicks is around 5 km^64^, that the communication range of whistling bottlenose dolphins is about 6 km^65^ and that whistles propagate farther than echolocation clicks^66^, in this study we assume a maximum detection range of about 6 km. This means that dolphins detected acoustically were over the plateau or slopes of Condor and Gigante seamounts (Fig. 1b,c).

### Statistical analysis

Because most months were only monitored once during the study period, we could not assess monthly or inter-annual variation in acoustic detections or foraging activity of dolphins. Instead, data were pooled by month of the year (hereafter called sampling periods) within each seamount, and sampling periods were treated.

A GLM model, with a binomial distribution and a logit link function, was used to investigate if dolphin detections varied between seamounts and sampling periods. The response variable was the proportion of dolphin positive hours per day (DPH), calculated as the number of hours with at least one dolphin detection divided by the number of hours recorded on that day, to account for variation in acoustic effort. A non-parametric pairwise Wilcoxon rank-sum test was used to detect differences in DPH between pairs of sampling periods. Dolphin foraging behaviour at seamounts was examined by calculating the proportion of foraging positive hours per day (FPH), i.e., the number of hours with at least one foraging signal divided by the number of hours with dolphin detections on that day. A GLM, with a binomial distribution and a logit link function, was used to explore the effect of seamount and sampling period in FPH.

Diel patterns in dolphin detections were examined with a GAMM model, with a binomial distribution and logit link function, using presence/absence of dolphin detections in each hourly record as the response variable. As the duration of day-night cycles varies considerably between summer and winter months, hours after the sunset was used as an explanatory variable. The local times of sunrise and sunset were obtained from the U.S. Naval Observatory Astronomical Applications Department database. Because dolphin detections in January-February 2011 and March 2008 showed a different day-night pattern from detections in the remaining sampling periods (Fig. 2), we included a different smoother for hours after sunset for each period (January-March and April-December). To account for differences in sampling effort per day, log of recording hours was used as an offset and an autoregressive moving average (ARMA) autocorrelation structure was included in the model to address the temporal dependence in the data^67^. Similar models were used to investigate diel patterns in dolphin acoustic behaviour, using presence/absence of different classes of signals as the response variable. A separate model was run for foraging (buzzes + bray calls), echolocation (clicks) and social (whistles + burst-pulsed) signals. A backward stepwise selection procedure was used to identify the best fitting model based on the Akaike Information Criterion (AIC) value and analysis of deviance. All statistical analyses were done in R^68^ using the ‘*mgcv’* R package^69^.

## Supplementary information

Supplementary Information.

## Data Availability

The datasets generated and/or analysed during this study can be made available upon request.
