# Supplementary material for: Temporal patterns in acoustic presence and foraging activity of oceanic dolphins at seamounts in the Azores
Source: Sci Rep. 2020 Feb 27;10:3610. doi: 10.1038/s41598-020-60441-4 (PMC7046721; doi:10.1038/s41598-020-60441-4)
Supplement: Supplementary file 1 — Supplementary Information. [file 41598_2020_60441_MOESM1_ESM.pdf]

Supplementary information

Temporal patterns in acoustic presence and foraging activity of oceanic dolphins at seamounts in the Azores

Irma Cascão[1*], Marc O. Lammers[2,3], Rui Prieto[1], Ricardo S. Santos[1], Mónica A. Silva[1,4]

[1] Marine and Environmental Sciences Centre (MARE), Institute of Marine Research (IMAR) and Okeanos R&D Centre, University of the Azores, Rua Frederico Machado 4, 9901-862 Horta, Portugal

[2] Hawaiian Islands Humpback Whale National Marine Sanctuary, National Oceanic and Atmospheric Administration (NOAA), Kihei, HI 96753, USA

[3] Oceanwide Science Institute (OSI), PO Box 61692, Honolulu, HI 96744, USA

[4] Biology Department, Woods Hole Oceanographic Institution, Woods Hole, MA 02543, USA

* Corresponding author (email: irma.cascao@gmail.com)

Supplementary tables

Supplementary Table S1. Analysis of deviance (ANOVA) table for the best fitted GLM model of dolphin positive hours (DPH) and of foraging positive hours (FPH). Significant term ($p<0.05$) is shown in bold. S. period - sampling period, *df* - degrees of freedom, *Dev.* - deviance, *Res. df* - residual df, *Res dev.* - residual deviance.

	DPH					FPH				
	df	*Dev.*	*Res. df*	*Res. dev.*	*p-value*	*df*	*Dev.*	*Res. df*	*Res. dev.*	*p-value*
NULL			688	208.10				682	35.80	
Seamount	1	0.001	687	208.10	0.976	1	2.129	681	33.67	0.145
S. period	13	60.509	674	147.59	**< 0.001**	13	5.020	668	28.65	0.975

Supplementary Table S2. Pairwise comparisons using Wilcoxon rank-sum test of dolphin positive hours (DPH) between pairs of sampling periods at Condor seamount. Significant terms ($p<0.05$) are shown in bold.

	Jan2011	Feb2011	Mar2008	Apr2008	Apr2010	May2008	May2010	Jun2010	Jul2010	Aug2010	Sep2010	Oct2010	Nov2010
Feb2011	1.000	-	-	-	-	-	-	-	-	-	-	-	-
Mar2008	1.000	1.000	-	-	-	-	-	-	-	-	-	-	-
Apr2008	**0.001**	**<0.001**	**<0.001**	-	-	-	-	-	-	-	-	-	-
Apr2010	1.000	1.000	1.000	**<0.001**	-	-	-	-	-	-	-	-	-
May2008	**<0.001**	**<0.001**	**<0.001**	1.000	**<0.001**	-	-	-	-	-	-	-	-
May2010	1.000	**0.028**	**0.022**	0.474	1.000	**<0.001**	-	-	-	-	-	-	-
Jun2010	**<0.001**	**<0.001**	**<0.001**	1.000	**<0.001**	**0.043**	0.749	-	-	-	-	-	-
Jul2010	**0.034**	**<0.001**	**<0.001**	1.000	**0.008**	**<0.001**	1.000	1.000	-	-	-	-	-
Aug2010	**0.017**	**<0.001**	**<0.001**	1.000	**0.003**	**0.002**	1.000	1.000	1.000	-	-	-	-
Sep2010	1.000	**0.021**	**0.008**	1.000	0.159	**0.002**	1.000	1.000	1.000	1.000	-	-	-
Oct2010	**<0.001**	**<0.001**	**<0.001**	1.000	**<0.001**	1.000	**0.007**	1.000	0.102	0.400	0.052	-	-
Nov2010	1.000	**0.014**	**0.006**	0.539	0.248	**<0.001**	1.000	0.575	1.000	1.000	1.000	**0.002**	-
Dec2010	1.000	0.050	**0.015**	0.100	0.840	**<0.001**	1.000	0.129	1.000	1.000	1.000	**<0.001**	1.000

Supplementary Table S3. Pairwise comparisons using Wilcoxon rank-sum test of dolphin positive hours (DPH) between pairs of sampling periods at Gigante seamount. Significant terms ($p<0.05$) are shown in bold.

	Jan2011	Feb2011	Mar2008	Apr2008	May2008	Jul2010	Aug2010	Sep2010	Oct2010	Nov2010
Feb2011	1.000	-	-	-	-	-	-	-	-	-
Mar2008	**0.036**	0.129	-	-	-	-	-	-	-	-
Apr2008	**0.024**	0.096	1.000	-	-	-	-	-	-	-
May2008	**<0.001**	**0.001**	1.000	1.000	-	-	-	-	-	-
Jul2010	1.000	1.000	0.592	1.000	**0.003**	-	-	-	-	-
Aug2010	**0.022**	0.058	1.000	1.000	0.287	1.000	-	-	-	-
Sep2010	**0.012**	0.102	1.000	1.000	1.000	1.000	1.000	-	-	-
Oct2010	**0.001**	**0.009**	1.000	1.000	1.000	0.111	1.000	1.000	-	-
Nov2010	1.000	1.000	0.094	0.415	**<0.001**	1.000	0.381	0.082	**0.015**	-
Dec2010	1.000	1.000	**0.020**	**0.048**	**<0.001**	1.000	**0.003**	**0.004**	**0.002**	1.000

Supplementary Table S4. ANOVA table for the best fitted GAMM model of diel patterns in dolphin detections and acoustic signals. Significant terms ($p<0.05$) are shown in bold. H after SS – hours after sunset.

**Models**	**Smooth terms**	*edf*	*F*	*p-value*
Dolphin detections	s(H after SS)$_{\text{Jan-Mar}}$	1.000	35.70	**< 0.001**
(n = 16,421)	s(H after SS)$_{\text{Apr-Dec}}$	7.114	69.91	**< 0.001**
Foraging signals	s(H after SS)	3.646	16.92	**< 0.001**
(n = 7,966)				
Echolocation clicks	s(H after SS)	6.226	24.44	**< 0.001**
(n = 7,966)				
Social signals	s(H after SS)	6.527	14.10	**< 0.001**
(n = 7,966)				
